# Evaluation of chemical components in Citri Reticulatae Pericarpium of different cultivars collected from different regions by GC–MS and HPLC

**DOI:** 10.1002/fsn3.569

**Published:** 2017-12-19

**Authors:** Meixia Luo, Hujie Luo, Pingjun Hu, Yiting Yang, Bo Wu, Guodong Zheng

**Affiliations:** ^1^ Key Laboratory of Molecular Target & Clinical Pharmacology School of Pharmaceutical Sciences and the Fifth Affiliated Hospital Guangzhou Medical University Guangzhou Guangdong China; ^2^ Infinitus (China) Company Limited Guangzhou Guangdong China

**Keywords:** Citri Reticulatae Pericarpium, cluster analysis, flavonoids, GC–MS, HPLC, volatile oil

## Abstract

To discriminate the feasible differences and find potential similarities and relationships of Citri Reticulatae Pericarpium (CRP), this work was accomplished by a comprehensive and reliable method using gas chromatography–mass spectrometer (GC–MS) to analyze the volatile oils and high‐performance liquid chromatography (HPLC) simultaneously to determine the contents of five bioactive flavonoids, namely hesperidin, nobiletin, 3,5,6,7,8,3′,4′‐heptamethoxyflavone, tangeretin, and 5‐hydroxy‐6,7,8,3′,4′‐pentamethoxyflavone in 25 batches of CRP samples of 10 cultivars collected from different regions in China. The GC–MS analyses indicated that 98 compounds were successfully identified from the volatile oils obtained and the major constituents of volatile oil are d‐limonene, γ‐terpinene, α‐pinene, linalool, and myrcene. Even 2‐(methylamino) benzoate was found in all cultivar samples harvested at maturation stage. Under the optimal condition, the quantitative analyses of five bioactive flavonoids were successfully performed by HPLC and hierarchical cluster analysis (HCA). Results showed significant differences among cultivars in the contents of five bioactive flavonoids mentioned earlier. The HCA and GC–MS results provided a convenient approach which might be applied for rapid similarity evaluation and also holds the potential for analysis of compounds present in other plants. Therefore, this work obtained offers scientific basis to control quality and develop medicinal value of the medicinal materials in CRP.

## 1 INTRODUCTION

Citri Reticulatae Pericarpium (CRP, Chenpi in Chinese), the dried ripe fruits peel of *Citrus reticulata* Blanco and its cultivars collected between September and December, has been broadly applied to a famous traditional Chinese medicine (TCM) and widely added to food as a condiment in China because of its different pharmacologic effects, low toxicity, and costs (Committee of National Pharmacopoeia, 2015; Duan, Guo, Liu, Liu, & Li, 2014; Shen, 2002). As a vital Chinese herbal medicine, CRP possesses various therapeutic activities, including strengthening spleen, promoting qi, eliminating dampness and phlegm, and so forth (Committee of National Pharmacopoeia, 2015; Xue & Xu, 2005). Dried tangerine or orange peels are widely distributed in different regions, such as Guangdong Province, Fujian Province, Sichuan Province, Zhejiang Province, Jiangxi Province, Hunan Province, and so forth. Guang Chenpi (*Citrus reticulata* “Chachi”), Chuan Chenpi (*C. reticulata* “Dahongpao”), Zhe Chenpi (*C. reticulata* “Unshiu”), and Jian Chenpi (*C. reticulata* “Tangerina”) are recorded by Chinese pharmacopeia. Among of the main Chenpi cultivars, the dried ripe pericarp of *C. reticulata* “Chachi,” mainly produced in Xinhui district of Guangdong Province in China, named Guang Chenpi (GCP) in Chinese, is viewed as famous drug of the region on account of its superior quality (Tan et al., 2015).

Phytochemical studies showed that abundant components are present in CRP, such as flavonoids, alkaloids, phenolic acids, and essential oils (EOs), among which flavonoids were considered to be the primary bioactive components (Zheng et al., 2013). Moreover, the major components of CRP are dietary flavonoids, which are generally categorized into two groups: flavanone glycosides (primarily hesperidin) and polymethoxylated flavones (PMFs, primarily nobiletin and tangeretin) (Ho & Kuo, 2014; Zeng, Dua, Chen, Li, & Liu, 2017). Currently, hesperidin is used as a chemical reference for quality control of CRP in the Chinese pharmacopeia because of its extremely high concentration (over 3%) (Committee of National Pharmacopoeia, 2015). Besides, EOs are the other principal pharmacological components of CRP (Qin et al., 2013). EOs extracted from plant materials, such as flowers, roots, bark, seeds, fruit peels, and wood, are subtle and aromatic. By report, several methods have been applied for extracting EOs, namely steam distillation (SD) and supercritical fluid extraction‐CO_2_ (SFE‐CO_2_), and SD as the common method is recorded on Chinese pharmacopeia, while several studies have quantified flavonoids in *Citrus* herbs using thin‐layer chromatography (TLC), HPLC–UV, HPLC–DAD, HPLC–ECD, HPLC–MS, and CE–ECD, primarily focusing on the determination of flavonoids in fruits, juices, or CRP of different *Citrus* species (Camarda, Di Stefano, Del Bosco, & Schillaci, 2007; Careri, Elviri, Mangia, & Musci, 2000; Liu et al., 2013; Peng, Liu, & Ye, 2006; Sahraoui et al., 2011; Wang & Luo, 1989; Zheng et al., 2009). In recent studies, lots of attention is being paid to flavonoids because of its anticancer, antioxidant, anticonvulsant, and anti‐inflammatory properties (Chang et al., 2015; Devi et al., 2015; Dimpfel, 2006; Fu et al., 2017), whereas few on volatile oils which have abundantly valid biological activities on CRP (Duan et al., 2016; Yi, Dong, Liu, Yi, & Zhang, 2015). So far, there were a group of analyses of flavonoids including constituents and contents, and the results indicated obvious differences in CP and GCP (Lin, Li, Ho, & Lo, 2012; Liu et al., 2013; Xing, Zhao, Zhang, & Li, 2017; Zhang et al., 2012; Zheng et al., 2009, 2013).

Modern pharmacology study has demonstrated that volatile oils play critical roles in certain allergy and antitussive, expectorant, and anti‐inflammatory effects (Wang et al., 2014). In addition, most reports on volatile oils components of Chenpi are focused on the comparison in citrus, such as Citri Reticulatae Pericarpium (CRP) and Citri Reticulatae Pericarpium Viride (CRPV), but few is centered around different cultivars (Chen & Cui, 1998; Gao, 2011; Hu et al., 2014; Mao, Ou, & Wang, 2015; Wang & Li, 2015; Yi et al., 2015). Gas chromatography coupled with mass spectroscopy (GC–MS) and high‐performance liquid chromatography (HPLC) with dual wavelength detection have become a reasonable and powerful approach for the qualitative and quantitative analyses in tangerine peels. However, to the best of our knowledge, no such study was yet reported about HPLC method coupled with GC–MS approach to evaluate and discriminate the quality of CRP effectively and comprehensively among different cultivars in order to ensure its superior clinical use.

**Figure 1:**
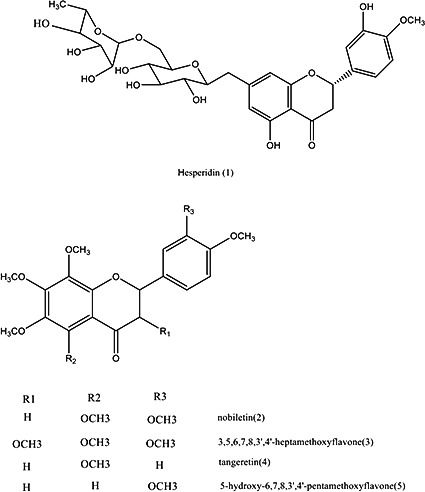
Chemical structure of bioactive flavonoids C1–C5

Thus, the objectives of this study were to develop a comprehensive, accurate, and reliable HPLC method for the simultaneous quantitative determination of five bioactive flavonoids (Figure 1), including hesperidin (C1), nobiletin (C2), 3,5,6,7,8,3′,4′‐heptamethoxyflavone (C3), tangeretin (C4), and 5‐hydroxy‐6,7,8,3′,4′‐pentamethoxyflavone (C5), as well as qualitative profiling of other secondary metabolites in 25 batches of tangerine peel samples of 10 cultivars collected from different major citrus‐producing areas in China, such as Guangdong Province, Guangxi Province, Sichuan Province, Fujian Province, Zhejiang Province, Jiangxi Province, Hubei Province, and Hunan Province. The reliability and adaptability of the method were verified by the determination of linear range, recovery, and reproducibility with CRP samples. And the results were evaluated and classified by hierarchical cluster analysis (HCA) based on the contents of five bioactive flavonoids. The results provide detailed information for identifying botanical origin, chemotaxonomic investigation of *Citrus* species, and potential perspective for quality control of complex matrices.

## 2 MATERIALS AND METHODS

### 2.1 Plant materials and chemicals

Twenty‐five samples including 10 different cultivars (collected between October 2013 and December 2015) were collected from different major citrus‐producing areas in China. The detailed information of the samples is presented in Table 1. Approximately 20 kg of fresh fruits was collected from each sampling area. Then, the citrus peels were removed and dried under the sun for about 5–7 days, which were used for testing the samples. And the voucher specimens, authenticated by Prof. Guodong Zheng, have been deposited at the Laboratory of Institute of Pharmaceutical Sciences, Guangzhou Medical University, Guangzhou University City, Guangdong Province, China.

**Table 1:** Sample information and the results of the volatile oil extraction (*n *=* *25)

Sample	Cultivars	Place of collection	Time of collection	Essential oil yield (g/kg)	Extract characters
S1	*Citrus reticulata* “Chachi”	Sanjiang Town, Xinhui District, Jiangmen City, Guangdong Province	2015/11/05	67.43 ± 0.08	Colorless
S2	*C. reticulata* “Chachi”	Sanjiang Town, Xinhui District, Jiangmen City, Guangdong Province	2015/12/23	62.94 ± 0.04	Colorless
S3	*C. reticulata* “Chachi”	Gujing Village, Xinhui District, Jiangmen City, Guangdong Province	2015/10/11	89.91 ± 0.35	Colorless
S4	*C. reticulata* “Chachi”	Gujing Village, Xinhui District, Jiangmen City, Guangdong Province	2010	5.84 ± 0.13	Pale yellow
S5	*C. reticulata* “Chachi”	Gujing Village, Xinhui District, Jiangmen City, Guangdong Province	2005	1.80 ± 0.05	Aqua
S6	*C. reticulata* “Chachi”	Dongjia Town, Xinhui District, Jiangmen City, Guangdong Province	2010	4.50 ± 0.04	Dark yellow
S7	*C. reticulata* “Chachi”	Dongjia Town, Xinhui District, Jiangmen City, Guangdong Province	2005	2.70 ± 0.02	Pale yellow
S8	*C. reticulata* “Chachi”	Tianlu Village, Xinhui District, Jiangmen City, Guangdong Province	2005	17.98 ± 0.05	Aqua
S9	*C. reticulata* “Chachi”	Shijian Town, Xinhui District, Jiangmen City, Guangdong Province	1995	7.19 ± 0.01	Yellow‐green
S10	*C. reticulata* “Chachi”	Meijiang Town, Xinhui District, Jiangmen City, Guangdong Province	1995	4.50 ± 0.02	Yellow‐green
S11	*C. reticulata* “Chachi”	Huaiji County, Zhaoqing City, Guangdong Province	2015/11/26	2.70 ± 0.01	Pale yellow
S12	*C. reticulata* “Chachi”	Longmen County, Huizhou City, Guangdong Province	2015/11/10	20.21 ± 0.09	Colorless
S13	*C. reticulata* “Unshiu”	Lingui County, Guilin City, Guangxi Zhuang Autonomous Region	2013/10/03	2.25 ± 0.02	Aqua
S14	*C. reticulata* “Erythrosa”	Shimen County, Changde City, Hunan Province	2014/11/30	2.25 ± 0.11	Yellow‐green
S15	*C. reticulata* “Erythrosa”	Shimen County, Changde City, Hunan Province	2015/10/21	2.02 ± 0.05	Green
S16	*C. reticulata* “Erythrosa”	Shimen County, Changde City, Hunan Province	2015/11/30	1.35 ± 0.01	Yellow‐green
S17	*C. reticulata* “Speciosa”	Dianjun District, Yichang City, Hubei Province	2015/10/11	0.90 ± 0.03	Yellow‐green
S18	*C. reticulata* “Speciosa”	Dianjun District, Yichang City, Hubei Province	2015/12/11	4.04 ± 0.02	Yellow‐green
S19	*C. reticulata* “Speciosa”	Pujiang County, Chengdu City, Sichuan Province	2015/10/15	3.59 ± 0.05	Aqua
S20	*C. reticulata* “Kinokuni”	Nanfeng County, Fuzhou City, Jiangxi Province	2015/10/10	9.00 ± 0.13	Aqua
S21	*C. reticulata* “Dahongpao”	Pujiang County, Chengdu City, Sichuan Province	2015/10/17	6.74 ± 0.05	Yellow‐green
S22	*C. reticulata* “Subcompressa”	Yongquan Town, Linhai County, Taizhou City, Zhejiang Province	2014/10/15	0.90 ± 0.06	Yellow‐green
S23	*C. reticulata C. reticulata* “Tangerina”	Dongzhang Town, Fuqing County, Fujian Province	2015/12/24	4.50 ± 0.04	Yellow‐green
S24	*Citrus reticulata* “Ponkan”	Yongchun County, Quanzhou City, Fujian Province	2015/12/07	0.315 ± 0.09	Yellow‐green
S25	*C. reticulata* “Shiyueju”	Huaiji County, Zhaoqing City, Guangdong Province	2015/11/26	38.25 ± 0.20	Pale green

The solvents, HPLC grade n‐hexane and acetonitrile, were purchased from Honeywell (America). Deionized water (18 mol/L^−1^·Ω) was prepared by distilled water through a Milli‐Q system (Millipore, Milford, MA, USA). The reference standards of flavonoids C1–C5 (hesperidin, nobiletin, 3,5,6,7,8,3′,4′‐heptamethoxyflavone, tangeretin, and 5‐hydroxy‐6,7,8,3′,4′‐pentamethoxyflavone) purchased from Must (Chengdu, China) were isolated and purified from “Chachi” CRP by conventional column chromatography, and their structures were identified by EI‐MS, ^1^HNMR, and ^13^C NMR in comparison with the data from the literature. Their purities were determined to be 98% based on HPLC analysis using a peak area normalization method.

### 2.2 Sample preparation for HPLC analysis

The tested samples were cut into smaller pieces and further ground into powder (100 mesh). Each sample powder (0.5 g) was weighed accurately and extracted with 50 ml methanol using ultrasonicator at room temperature for 30 min at 320 W. After that, the sample was filtered and the 10 ml volume of solution was set at 25 ml volumetric flask. The obtained solution was filtered through a 0.22‐μm filter membrane, and 20 μl of the filtrate was subjected to HPLC analysis.

### 2.3 Sample preparation for GC–MS analysis

The peels were manually removed, sun‐dried, and stored under dry conditions. Before extraction, the peels were powdered using a mill (Xuyang Equipment Manufacture Company, China) and passed through a 24 mesh sieve. Approximately 200 g of every sample powder we collected were swollen by soaking in 2,000 ml of distilled water for 12 hr prior to hydrodistillation for 5 hr using a standard extractor. Then, the EOs were prepared according to the standard method described in Chinese pharmacopeia (2015 version). The volatile oil was collected in a sterilized glass vial. Water was removed from the volatile oil using anhydrous sodium sulfate. The extraction yield was calculated (in milliliter of oil), was stored at 4°C in the dark, and then dissolved in n‐hexane prior to GC–MS analysis. EO yield (g/kg) was calculated with the following formula: EO yield (g/kg) = [mass of EOs obtained (g)/mass of dry matter (kg)].

### 2.4 Instruments and experimental conditions

GC–MS analysis was performed using an Agilent Technologies 7890A GC coupled with a fused silica capillary column (HP‐5MS, 0.25 mm × 30 m, film thickness 0.25 μm) and equipped with a 5975C MS detector (Agilent Technologies, Palo Alto, CA, USA). The column temperature was set at 60°C initially (maintained for 2 min), which was then increased to 80°C at a rate of 1°C/min for 10 min, subsequently raised to 25°C at a rate of 5°C/min, and finally to 300°C at a rate of 20°C/min for 1 min. The inlet temperature was 270°C. The carrier gas was helium with a constant flow rate of 1.0 ml/min in a split ratio of 10:1 and the injection volume was 5 μl. To the experimental conditions of the mass spectrometer, electron impact (EI+) mass spectra were operated at 70 eV. Injector and detector temperatures were set at 270°C, respectively. Scan at 0.2 scan/s from *m*/*z* 30 to 550 amu and solvent was delayed by 4 min. All the data analyses were carried out on a personal computer. The library searches and spectral matching of the resolved pure components were conducted on the NIST (National Institute of Standards and Technology) 08s.L MS database.

### 2.5 HPLC analysis

The flavonoids were detected at 283 nm and 330 nm by the UV detector using an Agilent 1260 Series HPLC system (Shimadzu Corporation, Japan). The compounds were separated on a Dikma Diamonsil C18 column (4.6 mm × 250 mm i.d., 5 μm) and at a column temperature of 25°C. The solvent system consisted of acetonitrile (B) and water (A) with the following gradient elution program: 0 min 85% A + 15% B; 15 min 60% A + 40% B; 35 min 50% A + 50% B; 40 min 25% A + 75% B; 50 min 15% A + 85% B. The mobile phase conditions were modified from a previously reported method (Zheng et al., 2009). The flow rate was 1.0 ml/min, and the injection volume of the sample was 20 μl. The effluent was monitored by UV detection at 283 nm for compound C1 and 330 nm for compounds C2–C5.

### 2.6 Establishment of calibration curves

To build the calibration curves, the mixed standard stock solution was prepared by dissolving the reference compounds (C1–C5) in methanol with the final concentrations of each compound at 483.6, 203.2, 160.8, 163.6, and 160.8 μg/ml, respectively. Working standard solutions containing each of the five compounds were prepared by diluting the stock solutions with methanol to a series of proper concentrations. Resulting solutions were filtered through a 0.22‐μm nylon syringe filters, and aliquots of 20 μl were injected in the chromatographic system for analyses. These solutions were stored at 4°C for further HPLC analysis. The standard solutions were analyzed in triplicates, and peak areas were used as analytical signal. The calibration curves were constructed by plotting the peak areas versus the concentrations of standards.

### 2.7 Method validation

The method was validated for linearity, sensitivity, repeatability, and accuracy. The limits of detection (LOD) and limits of quantification (LOQ) under the present conditions were determined at an S/N (signal to noise) of about 3 and 10, respectively. Intraday variations were chosen to determine the precision of the developed assay. For intraday variability test, three different amounts (high, middle, and low levels) of reference compounds were analyzed for six replicates within 1 day. Variations were expressed by the relative standard deviation (RSD) of the data. Recovery was used to evaluate the accuracy of the method. Six different amounts of the standard solutions were added to sample S1, and the recovery was measured in triplicate. For comparison, unspiked S1 sample was concurrently prepared and analyzed. The recovery was calculated as follows: Recovery (%) = (Detection − Original Amount)/Addition × 100%. For the stability test, the sample solution was analyzed using the established method at 0, 2, 4, 6, 8, 10, 12, 24, and 48 hr, respectively, the peak areas of five analytes were recorded, and the RSD of peak areas at different times were calculated.

### 2.8 Hierarchical cluster analysis

HCA is a statistical approach to distinguish homogeneous groups of cases based on tested characteristics. HCA is carried out to study the distances between pairs of samples, in order to highlight groupings between them through the Euclidean distance algorithm using single linkage clustering. When distance between samples is relatively small, it implies that samples are similar. Therefore, the contents of the five analytes were defined as five characteristics in the analysis to differentiate and classify the 25 CRP accessions examined during experiment. HCA of samples were performed by SPSS software (SPSS 16.0 for Windows, SPSS Inc., USA).

## 3 RESULTS

### 3.1 Analytical method validation of HPLC

**Figure 2:**
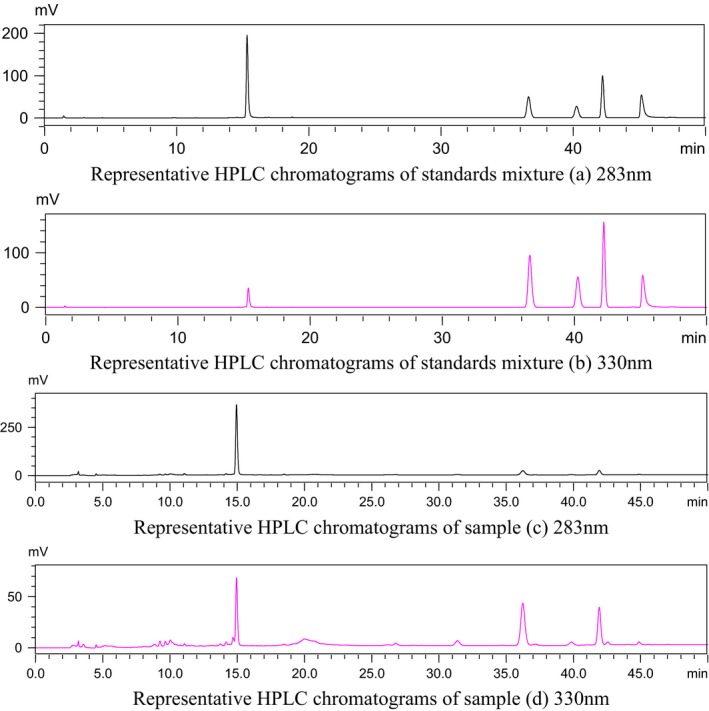
HPLC chromatograms of standard mixture (a) and sample (b). The numbers indicate bioactive flavonoids C1–C5

To establish informative and reliable HPLC condition of tangerine peels, detailed information regarding calibration curves, linear ranges, LOD, and LOQ is displayed in Table 2. For all the examined compounds, all five calibration curves exhibited good linearity (*R* ^2^ > .9990), the intraday precisions, repeatability, stability, recovery calculated as relative standard deviation (RSD) were all <3%, and the accuracy ranged from 98.81% to 100.08% which are displayed in Tables 3, 4, 5, respectively. The results revealed the developed method was applied to the determination of the five flavonoids in samples collected from different regions in China. Representative HPLC chromatograms of standard mixture and sample under the optimized conditions are shown in Figure 2.

**Table 2:** Calibration curve data for reference compounds 1–5 (*n *=* *3)

Compounds	Regression equation (*y* = *ax* + *b*)^a^	*R* ^2^	Linear range (μg/ml)	LOD (μg/ml)	LOQ (μg/ml)
1	*y *=* *34,031*x* + 75,594	.9993	48.36–338.52	0.02	0.05
2	*y *=* *76,076*x* + 55,702	.9993	4.06–101.60	0.02	0.06
3	*y *=* *56,695*x* + 1699	.9996	0.08–21.00	0.02	0.08
4	*y *=* *83,002*x* + 19,904	.9996	3.27–81.80	0.01	0.04
5	*y *=* *59,354*x* − 6578	.9990	0.08–5.00	0.02	0.08

a *y* and *x* denote the peak area and corresponding injection concentration (μg/ml), respectively. *a* and *b* denote the slope and intercept of the regression line, respectively.

**Table 3:** Intraday precision of the developed method (*n *=* *6)

Compounds	Concentration (μg/ml)	Intraday		
		Detected^a^ (μg/ml)	Accuracy (%)	RSD (%)
1	290.16	291.61 ± 0.50	100.50	0.17
	338.52	337.32 ± 0.44	99.65	0.13
	483.60	510.07 ± 1.04	105.47	0.20
2	121.92	127.31 ± 0.02	104.42	0.02
	142.24	147.25 ± 0.19	103.52	0.13
	203.20	221.49 ± 1.14	109.00	0.50
3	96.48	103.73 ± 0.03	107.51	0.03
	112.56	119.89 ± 0.10	106.51	0.08
	160.80	173.19 ± 0.57	107.71	0.33
4	98.16	100.82 ± 0.02	102.71	0.02
	114.52	116.60 ± 0.10	101.82	0.09
	163.60	176.12 ± 0.59	107.65	0.34
5	96.48	101.64 ± 0.21	105.35	0.21
	112.56	117.81 ± 0.06	104.66	0.05
	160.80	176.48 ± 0.19	109.75	0.11

a Data are represented as the mean ± *SD*.

**Table 4:** Analysis repeatability and stability of the developed method

Compounds	Repeatability (*n *=* *6)			Stability (*n *=* *8)		
	RT (min)^a^	Content (mg/g)^a^	RSD (%)	RT (min)^a^	Content (mg/g)^a^	RSD (%)
1	15.17 ± 0.29	3.03 ± 0.06	2.07	15.10 ± 0.34	3.02 ± 0.03	1.03
2	36.49 ± 0.47	0.46 ± 0.01	1.97	36.37 ± 0.55	0.47 ± 0.01	0.95
3	40.17 ± 0.46	0.04 ± 0.01	2.727	39.97 ± 0.52	0.05 ± 0.01	2.53
4	42.09 ± 0.28	0.30 ± 0.01	2.25	42.00 ± 0.32	0.31 ± 0.01	0.81
5	45.00 ± 0.16	0.03 ± 0.01	2.74	44.95 ± 0.18	0.04 ± 0.01	2.53

a Data are represented as the mean ± *SD*.

**Table 5:** Recovery data of the developed method (*n *=* *6)

Compounds	Concentration of analyte			Recovery (%)^a^	RSD (%)
	Original (μg/ml)^a^	Spiked (μg/ml)	Found (μg/ml)^a^		
1	70.89 ± 2.06	87.05	158.01 ± 2.90	100.08 ± 1.54	1.54
2	9.02 ± 0.26	36.58	45.24 ± 0.77	99.03 ± 2.12	2.14
3	1.69 ± 0.06	28.94	30.63 ± 0.34	99.98 ± 1.21	1.21
4	12.08 ± 0.32	29.45	41.18 ± 0.62	98.81 ± 2.61	2.64
5	1.35 ± 0.037	28.94	30.20 ± 0.52	99.67 ± 1.78	1.79

a Data are represented as the mean ± *SD*.

### 3.2 Quantitative analysis of samples in HPLC

The developed analytical method was subsequently applied to the simultaneous determination of above five flavonoids in 25 batches of CRP collected from different major citrus*‐*producing regions in China. The contents of oil and five analytical compounds presented as the average value ± *SD* are summarized in Table 6. The data indicated that the content of each flavonoid varied significantly among the different cultivars and regions. Among the cultivars tested, the content of hesperidin (compound 1) is the richest in 25 samples varied from 16.6 ± 0.01 to 79.6 ± 0.04 mg/g, but the content of hesperidin was found to be much less in the peel of *Citrus reticulata* “Chachi” than in other cultivars. And nobiletin and tangeretin are the major PMF components in CRP. These results are in line with the results of Zheng et al. (2009) and Liu et al. (2013).

**Table 6:** Contents (mg/g, *n *=* *3)^a^ of five bioactive flavonoids in CRP samples collected from different regions in China

Sample	Cultivars	Place of collection	Time of collection	Hesperidin	Nobiletin	3,5,6,7,8,3′,4′‐Heptamethoxyflavone	Tangeretin	5‐Hydroxy‐6,7,8,3′,4′‐pentamethoxyflavone
S1	*Citrus reticulata* “Chachi”	Sanjiang Town, Xinhui District, Jiangmen City, Guangdong Province	2015/11/05	24.34 ± 0.03	2.92 ± 0.00	0.27 ± 0.00	1.81 ± 0.00	0.14 ± 0.00
S2	*C. reticulata* “Chachi”	Sanjiang Town, Xinhui District, Jiangmen City, Guangdong Province	2015/12/23	16.60 ± 0.01	2.22 ± 0.00	0.16 ± 0.00	1.41 ± 0.00	0.10 ± 0.00
S3	*C. reticulata* “Chachi”	Gujing Village, Xinhui District, Jiangmen City, Guangdong Province	2015/10/11	29.61 ± 0.06	4.09 ± 0.01	0.38 ± 0.00	2.73 ± 0.00	0.20 ± 0.00
S4	*C. reticulata* “Chachi”	Gujing Village, Xinhui District, Jiangmen City, Guangdong Province	2010	25.33 ± 0.01	3.33 ± 0.00	0.21 ± 0.00	2.70 ± 0.00	0.23 ± 0.00
S5	*C. reticulata* “Chachi”	Gujing Village, Xinhui District, Jiangmen City, Guangdong Province	2005	21.82 ± 0.02	3.05 ± 0.01	0.28 ± 0.00	2.51 ± 0.00	0.22 ± 0.00
S6	*C. reticulata* “Chachi”	Dongjia Town, Xinhui District, Jiangmen City, Guangdong Province	2010	29.54 ± 0.02	3.34 ± 0.00	0.26 ± 0.00	2.84 ± 0.00	0.24 ± 0.00
S7	*C. reticulata* “Chachi”	Dongjia Town, Xinhui District, Jiangmen City, Guangdong Province	2005	27.93 ± 0.01	3.88 ± 0.00	0.26 ± 0.00	3.04 ± 0.00	0.27 ± 0.00
S8	*C. reticulata* “Chachi”	Tianlu Village, Xinhui District, Jiangmen City, Guangdong Province	2005	29.33 ± 0.01	3.76 ± 0.00	0.30 ± 0.00	2.98 ± 0.00	0.27 ± 0.00
S9	*C. reticulata* “Chachi”	Shijian Town, Xinhui District, Jiangmen City, Guangdong Province	1995	32.01 ± 0.01	3.59 ± 0.00	0.23 ± 0.00	3.02 ± 0.00	0.30 ± 0.00
S10	*C. reticulata* “Chachi”	Meijiang Town, Xinhui District, Jiangmen City, Guangdong Province	1995	36.94 ± 0.00	3.76 ± 0.00	3.96 ± 0.00	3.03 ± 0.00	0.27 ± 0.00
S11	*C. reticulata* c	Huaiji County, Zhaoqing City, Guangdong Province	2015/11/26	47.99 ± 0.02	5.09 ± 0.00	0.04 ± 0.00	2.13 ± 0.00	0.38 ± 0.00
S12	*C. reticulata* “Chachi”	Longmen County, Huizhou City, Guangdong Province	2015/11/10	38.32 ± 0.00	3.70 ± 0.00	0.27 ± 0.00	3.11 ± 0.00	0.32 ± 0.00
S13	*C. reticulata* “Unshiu”	Lingui County, Guilin City, Guangxi Zhuang Autonomous Region	2013/10/03	57.07 ± 0.01	0.40 ± 0.00	0.69 ± 0.00	0.19 ± 0.00	0.02 ± 0.00
S14	*C. reticulata* “Erythrosa”	Shimen County, Changde City, Hunan Province	2014/11/30	65.36 ± 0.01	0.32 ± 0.00	0.34 ± 0.00	0.17 ± 0.00	0.02 ± 0.00
S15	*C. reticulata* “Erythrosa”	Shimen County, Changde City, Hunan Province	2015/10/21	59.62 ± 0.01	0.37 ± 0.00	0.47 ± 0.00	0.18 ± 0.00	0.02 ± 0.00
S16	*C. reticulata* “Erythrosa”	Shimen County, Changde City, Hunan Province	2015/11/30	57.61 ± 0.05	0.27 ± 0.00	0.38 ± 0.00	0.11 ± 0.00	0.01 ± 0.00
S17	*C. reticulata* “Speciosa”	Dianjun District, Yichang City, Hubei Province	2015/10/11	68.07 ± 0.01	0.60 ± 0.00	0.35 ± 0.00	0.30 ± 0.00	0.03 ± 0.00
S18	*C. reticulata* “Speciosa”	Dianjun District, Yichang City, Hubei Province	2015/12/11	73.29 ± 0.05	0.46 ± 0.00	0.29 ± 0.00	0.24 ± 0.00	0.02 ± 0.00
S19	*C. reticulata* “Speciosa”	Pujiang County, Chengdu City, Sichuan Province	2015/10/15	72.76 ± 0.03	0.26 ± 0.00	0.32 ± 0.00	0.14 ± 0.00	0.02 ± 0.00
S20	*C. reticulata* “Kinokuni”	Nanfeng County, Fuzhou City, Jiangxi Province	2015/10/10	44.39 ± 0.02	6.91 ± 0.00	0.18 ± 0.00	4.82 ± 0.00	0.69 ± 0.00
S21	*C. reticulata* “Dahongpao”	Pujiang County, Chengdu City, Sichuan Province	2015/10/17	60.52 ± 0.00	5.75 ± 0.00	0.10 ± 0.00	2.54 ± 0.00	0.43 ± 0.00
S22	*C. reticulata* “Subcompressa”	Yongquan Town, Linhai County, Taizhou City, Zhejiang Province	2014/10/15	79.65 ± 0.04	0.22 ± 0.00	0.37 ± 0.00	0.11 ± 0.00	0.02 ± 0.00
S23	*C. reticulata* “Tangerina”	Dongzhang Town, Fuqing County, Fujian Province	2015/12/24	49.59 ± 0.01	4.66 ± 0.01	0.11 ± 0.00	2.51 ± 0.00	0.34 ± 0.00
S24	*C. reticulata* “Ponkan”	Yongchun County, Quanzhou City, Fujian Province	2015/12/07	44.91 ± 0.01	4.77 ± 0.00	0.04 ± 0.00	3.53 ± 0.00	0.84 ± 0.00
S25	*C. reticulata* “Shiyueju”	Huaiji County, Zhaoqing City, Guangdong Province	2015/11/26	60.58 ± 0.07	3.83 ± 0.00	0.04 ± 0.00	3.60 ± 0.00	0.15 ± 0.00

### 3.3 Quality assessment of CRP by HCA

**Figure 3:**
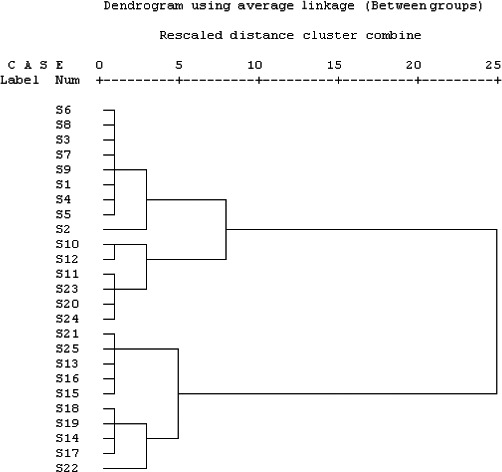
Dendrogram of hierarchical cluster analysis for 25 Citri Reticulatae Pericarpium samples in HPLC collected from different major citrus*‐*producing areas in China. Rescaled distance cluster combine was selected as a measurement. Citri Reticulatae Pericarpium samples were divided into two main clusters

To evaluate the change in similarity of five flavonoids displayed by the 25 CRP samples of different cultivars, an HCA based on the major five flavonoids was performed. The HCA is a multivariate procedure that allows the classification of variables into groups based on Euclidean distances between cases. The dendrogram of the 25 CRP samples is shown in Figure 3. The HCA results demonstrated a better hierarchical classification with all the samples well grouped according to their species. Obviously, as given in Figure 3, CRP samples were apparently classified as one of two groups so that this analysis provided further profound understanding of the distribution of the multiple chemical compounds in CRP. Two major clusters, viz., clusters 1 and 2, can be found in Figure 3. Cluster 1 was composed of 15 samples, while cluster 2 was composed of 10 samples. Although the concentrates of the samples S1–S12 (*C. reticulata* “Chachi”) from Guangdong Province are distinguishing, they could be classified into one cluster. In other words, the chemical ingredients of *C. reticulata* “Chachi” are similar. It can be seen that cluster 2 includes *C. reticulata* “Speciosa,” *C. reticulata* “Dahongpao,” *C. reticulata* “Subcompressa,” *C. reticulata* “Erythrosa,” *C. reticulata* “Unshiu,” and *C. reticulata* “Shiyueju,” which indicates that the six species possess the similarities and relationships to some extent. In addition, it can be useful for us to choose the appropriate dried tangerine peels with desired content of components as well as to choose alternate cultivars with more similarity in the absence of a desired variety at the time of requirement.

### 3.4 Global yields of volatile oil in CRP

In the experiment, EOs were extracted from a total of 15 batches of 10 varieties of dried tangerine peels by using extraction method of EOs in Chinese pharmacopeia (2015 edition). The contents of oil are presented as the average value ± *SD* (W/W). The EOs extraction yield results acquired among the 25 CRP samples based on a dry weight are present in Table 1. Obviously, as can be seen that a total of EOs obtained were transparent and oily liquid with a rich smell, whose extraction yield approximately ranges from 0.90 to 67.43 (g/kg). And there existed significant differences in EOs contents and their color, which may be affected by the cultivars and environmental factors, such as storage conditions, time of collection, and preservation. According to Table 1, it is noticeable that *C. reticulata* “Chachi” which is mainly produced in Xinhui, Guangdong are rich in EOs except for *C. reticulata* “Shiyueju,” even the highest EOs yield reach up to 67.43 g/kg, whereas the lowest is only 0.90 g/kg recorded for the *C. reticulata* “Speciosa” picked from Dianjun District, Yichang City, Hubei Province and *C. reticulata* “Subcompressa” collected from Yongquan Town, Linhai County, Taizhou City, Zhejiang Province, respectively. In addition, the Table 1 showed an obvious difference in the EOs extraction ratio of *C. reticulata* “Chachi” produced in diverse regions, and found that the extracting rate of *C. reticulata* “Chachi” producing in Xinhui district, Guangdong Province is higher than other areas in Guangdong Province. Moreover, it was discovered that the less EOs contents, the deeper the color in this test, and a tentative inference on this result is that the color of EOs may have certain correlation with oil content.

### 3.5 Typical total ion chromatogram of volatile oil in CRP

**Figure 4:**
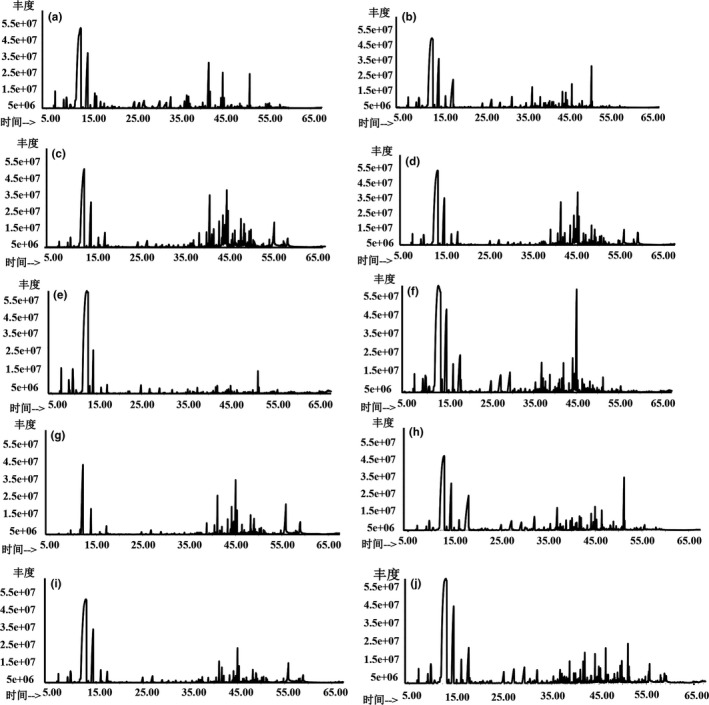
Total ion chromatography of volatile oil from CRP. Temperature programmed gas chromatography (TPGC) and electron impact (EI) ion source were used for the analysis of complex samples that we described in the Materials and Methods. Constituents were identified by comparison of authentic compounds with reference spectra in the computer library (NIST 08s.L MS database) and confirmed by comparison of those authentic compounds with data in literature. CRP of (a) *Citrus reticulata* “Chachi,” (b) *C. reticulata* “Dahongpao,” (c) *C. reticulata* “Subcompressa,” (d) *C. reticulata* “Erythrosa,” (e) *C. reticulata* “Shiyueju,” (f) *C. reticulata* “Kinokuni,” (g) *C. reticulata* “Speciosa,” (h) *C. reticulata* “Tangerina,” (i) *C. reticulata* “Unshiu,” and (j) *C. reticulata* “Ponkan”

**Figure 5:**
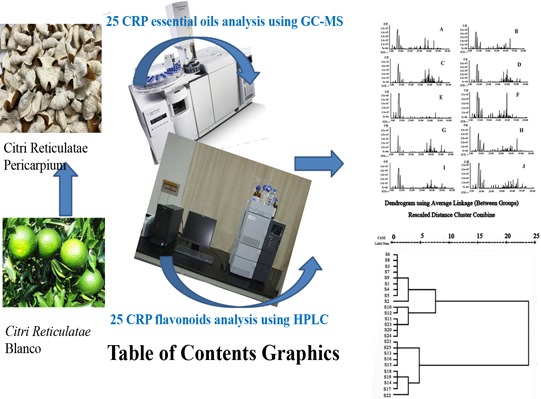
Graphic for table of contents

GC–MS analysis has been the most popular and powerful assistant technique to identify volatile constituents of EOs. In this study, methanol extracts of CRP samples collected from different citrus‐producing areas in China were analyzed by GC–MS analysis under optimal conditions. A total of typical total ion chromatograms (TICs) from CRP samples obtained are shown in Figure 4, which exhibits the differences in TICs of the volatile oils of CRP samples among different cultivars collected from different major citrus‐producing areas in China. Apparently, as can be seen, each TIC is completely complex and diverse analytic system. Additionally, from Figure 1, there are significant differences on the peak height of the same retention time in CRP samples. It seems that different peak height is on behalf of the concentration of chemical constituent, and with all the spectra available, the chromatograms of several chief peaks can be easily detected. Then, the higher peak cluster represents the stronger content of components. Eventually, a library search was carried out for all the peaks using the NIST 08 s.L MS database Figure 5.

### 3.6 Comparative analysis of volatile components in CRP

Using GC–MS analysis, the quantity of the constituents tested was reach up to 98 with pretty good match which were identified via similarity comparison with compounds of database NIST 08 s.L MS database in this work. The 98 chemical compositions which have more than 90% similarity were identified for the EO from CRP peels extracted by SD, demonstrated in Table 7. For example, d‐limonene, γ‐terpinene, and 2‐(methylamino) benzoate were confirmed at 12.7, 14.4, and 41.7 min, respectively, by comparison of credible components with reference spectra in the computer library NIST 08 s.L MS database and identified by comparison of those authentic compounds with data in literature. Moreover, from Table 7, we have observed that the main constituents of the volatile oil of tangerine peels were almost monoterpenes and sesquiterpenes. Monoterpenes were d‐limonene, γ‐terpinene, myrcene, camphene, terpinolene, α‐pinene, and α‐phellandrene. Sesquiterpenes were elemene, α‐farnesene, α‐cubebene, valencene, α‐gurjunene, α‐caryophyllene, (+)‐aromadendrene, and aristolene. In addition, the fatty acid principally include (Z,Z)‐9,12‐tadecadienoic acid, (Z,Z,Z)‐9,12,15‐tadecatrienoic acid, octadecanoic acid, and hexadecanoic acid. Immediately following, gas chromatography area normalization method for the determination of the relative content of each component was used. The relative percentage concentrations of the principal chromatographic peak were measured with peak area normalization, which was summarized in Table 8. The types and contents of volatile oil components in part are similar, but they have certain differences in CRP from various regions. The data in Table 8 visibly indicated that the content of each volatile constituent is varied apparently among the diverse cultivars and regions, particularly among different disparate cultivars. Although d‐limonene (24.54%–73.09%) is the most ample volatile component, γ‐terpinene (3.69%–17.71%) the second in all samples obtained in CP, their percentage was diversified, based on the origins of the plant which are important index of volatile oil, in accordance with the literature (Duan et al., 2016; Zhou, Huang, Mo, & Liao, 2009). Therefore, the content differences of volatile constituents may result in various pharmacological effects of CP. Moreover, the content of d‐limonene was found to be much higher in the peel of *Citrus reticulata* “Shiyueju” than in other cultivars examined. On the contrary, the content of γ‐terpinene was discovered to be lower in the CRP cultivars. In short, the results of the components of the peel oil of CRP gained from this study are nearly in accordance with other prior researches. Furthermore, what is noteworthy is that in the whole examined, the percentage of 2‐dimethylamino methyl benzoate, the possessed chemical component is varied completely. The cultivars of GCP cultivated in Guangdong Province contain plentiful of specific component of 2‐dimethylamino‐methyl benzoate. Similarly, S9 and S12 have a considerable high concentration severally. Nevertheless, what is not consistent with previous conclusion (Zhou et al., 2009), was recorded that it existed only in the GCP. In summary, the data displayed crucial variations in the content of these EO constituents in the samples tested from various regions in China. Therefore, a significant difference in chemical components of the EOs might also be attributed to various factors, for instance, growth environment, genetic source, and geographical and environmental conditions, particularly genetic source.

**Table 7:** Volatile compounds of essential oils of CRP studied

Peak number	Retention time	Compounds	Molecular formula	Relative molecular mass
1	6.942	α‐Pinene	C_10_H_16_	136
2	7.612	Camphene	C_10_H_16_	136
3	8.657	α‐Phellandrene	C_10_H_16_	136
4	8.687	Sabinene	C_10_H_16_	136
5	8.912	β‐Pinene	C_10_H_16_	136
6	9.53	Myrcene	C_10_H_16_	136
7	10.319	Octana	C_8_H_16_O	128
8	11.168	α‐Terpinene	C_10_H_16_	136
9	11.536	1‐Methyl‐4‐(1‐methylethyl)benzene	C_10_H_14_	134
10	12.717	d‐Limonene	C_10_H_16_	136
11	13.144	Benzylcarboxaldehyde	C_8_H_8_O	120
12	13.304	13,7‐dimethyl‐3,6‐Octatriene	C_10_H_16_	136
13	13.316	Ocimene	C_10_H_16_	136
14	14.355	γ‐Terpinene	C_10_H_16_	136
15	14.462	*N*‐Methylaniline	C_7_H_9_N	107
16	15.043	1‐Octanol	C_8_H_16_O	130
17	15.969	Terpinolene	C_10_H_16_	136
18	16.064	1‐Methyl‐4‐(1‐methylethylidene)cyclohexene	C_10_H_16_	136
19	16.337	1‐Methyl‐4‐(1‐methylethenyl)‐benzene	C_10_H_12_	132
20	16.492	Benzoic acid methyl ester	C_8_H_8_O_2_	136
21	17.536	Linalool	C_10_H_18_O	132
22	17.75	1‐Nonanal	C_9_H_18_O	142
23	18.124	1,3,8‐p‐Menthatriene	C_10_H_14_	134
24	18.622	Bicyclo[2.2.1]heptan‐2‐ol,1,3,3‐trimethyl‐	C_10_H_18_O	154
25	18.711	3,5,5‐Trimethyl‐2‐cyclohexen‐1‐one;	C_9_H_14_O	138
26	19.821	Limonene oxide	C_10_H_16_O	152
27	19.21	2‐Cyclohexen‐1‐ol, 1‐methyl4‐(1‐methylethyl)‐trans‐	C_10_H_18_O	154
28	20.949	Campho	C_10_H_16_O	152
29	21.406	1‐Methyl‐4‐(1‐methylethenyl) cyclohexanol	C_10_H_18_O	154
30	21.982	Citronella	C_10_H_18_O	154
31	23.667	Borneol	C_10_H_18_O	154
32	24.932	4‐Terpineol	C_10_H_18_O	154
33	27.163	α‐Terpineol	C_10_H_18_O	154
34	29.276	Decanal	C_10_H_20_O	156
35	34.03	*cis*‐Carveol	C_10_H_16_O	152
36	31.383	Neml	C_10_H_18_O	154
37	31.941	Citronellol	C_10_H_20_O	156
38	32.956	Carvone	C_10_H_14_O	150
39	33.751	3‐Methyl‐6‐(1‐methylethyl)‐2‐cyclohexen‐1‐one	C_10_H_16_O	152
40	34.019	Geraniol	C_10_H_18_O	154
41	35.2	Citral	C_10_H_16_O	152
42	35.354	4‐(1‐Methylethenyl)‐1‐cyclohexene‐1‐carboxaldehyde	C_10_H_14_O	150
43	35.461	1‐Cyclohexene‐1‐carboxaldehyde,4‐(1‐methylethyl)‐	C10H16O	152
44	36.054	Nonanoic acid	C_9_H_18_O_2_	158
45	36.268	2‐Methyl‐5‐(1‐methylethyl)phenol	C_10_H_14_O	150
46	36.761	Thymol	C_10_H_14_O	150
47	37.004	Perillyl alcohol	C_10_H_16_O	152
48	37.129	3‐Methyl‐4‐isopropylphenol	C_10_H_14_O	150
49	37.532	2‐Methoxy‐4‐vinylphenol	C_9_H_10_O_2_	150
50	37.71	Undecanal	C_11_H_22_O	170
51	38.161	2,4‐Decadienal	C_10_H_16_O	152
52	38.363	(R)‐(+)‐Citronellic acid	C_10_H_18_O_2_	170
53	38.874	Methyl 2‐aminobenzoate	C_8_H_9_NO_2_	151
54	39.265	2,4,6‐Trichlorophenol	C_6_H_3_Cl_3_O	197
55	39.307	α‐Cubebene	C_15_H_24_	204
56	39.503	Eugenol	C_10_H_12_O_2_	164
57	39.693	2,6‐Octadiene,2,6‐dimethyl‐	C_10_H_18_	138
58	40.043	*cis*‐3,7‐Dimethyl‐2,6‐octadien‐1‐ol–acetate	C_12_H_20_O_2_	196
59	40.215	Benzene,4‐ethenyl‐1,2‐dimethoxy‐	C_10_H_12_O_2_	164
61	40.458	Copaene	C_15_H_24_	204
62	40.583	Decanoic acid	C_10_H_20_O_2_	172
63	40.868	*trans*‐3,7‐Dimethyl‐2,6‐octadien‐1‐yl ethanoate	C_12_H_20_O_2_	196
64	41.657	2‐(Methylamino)benzoate	C_9_H_11_NO_2_	165
65	41.96	Cyclodecane	C_10_H_20_	140
66	41.978	Dodecanal	C_12_H_24_O	184
67	42.999	Isovaleric acid octyl ester	C_13_H_26_O_2_	214
68	43.301	α‐Caryophyllene	C_15_H_24_	204
69	43.45	(+)‐Aromadendrene	C_15_H_24_	204
70	43.883	Undecanoic acid	C_11_H_22_O_2_	186
71	44.138	Bicyclosesquiphellandrene	C_15_H_24_	204
72	44.951	α‐Farnesene	C_15_H_24_	204
73	45.527	Methyl laurate	C_13_H_26_O_2_	214
74	45.913	α‐Calacorene	C_15_H_20_	200
75	46.382	Elemene	C_15_H_24_	204
76	45.682	α‐Gurjunene	C_15_H_24_	204
77	45.8	Valencene	C_15_H_24_	204
78	45.919	Aristolene	C_15_H_24_	204
79	46.512	Nemlidol	C_15_H_26_O	222
81	46.892	Dodecanoic acid	C_12_H_24_O_2_	200
82	47.142	(−)‐Globulol	C_15_H_26_O	222
83	47.883	Tetradecanal	C_14_H_28_O	212
84	48.097	Longifolene	C_15_H_24_	204
85	48.887	α‐Eudesmol	C_15_H_26_O	222
86	49.379	Spathulenol	C_15_H_24_O	202
87	51.617	1,4‐Dimethyl‐7‐isopropylazulene	C_15_H_18_	198
88	51.724	Phenanthrene/Anthracene	C_14_H_10_	178
89	52.27	Nootkatone	C_15_H_22_O	218
90	54.774	Hexadecanoic acid, methyl ester	C_17_H_34_O_2_	270
91	54.952	Tetradecanoic acid	C_14_H_28_O_2_	228
92	55.635	Hexadecanoic acid	C_16_H_32_O_2_	256
93	56.116	Octadecanoic acid	C_18_H_36_O_2_	284
94	57.991	9,12‐Octadecadienoic acid(Z,Z)‐,methyl ester	C_19_H_34_O_2_	294
95	58.092	(Z,Z,Z)‐9,12,15‐Octadecatrienoic acid methyl ester	C_19_H_32_O_2_	292
96	58.870	(Z,Z,Z)‐9,12,15‐Octadecatrienoic acid	C_18_H_30_O_2_	278
97	58.976	(Z,Z)‐9,12‐Octadecadienoic acid	C_18_H_32_O_2_	280
98	59.202	Ethyl linoleate	C_20_H_36_O_2_	308

**Table 8:** Main components’ area normalization results (*n *=* *25)

Sample NO Peak NO	Relative percentage content %											
	1	5	6	8	10	14	21	30	32	34	36	64
S1	2.49	1.96	3.01	0.08	64.64	16.86	0.18	0.04	0.61	0.26	0.01	1.82
S2	2.35	1.98	2.97	0.23	63.80	17.57	0.20	0.10	0.29	0.19	0.00	0.83
S3	2.61	2.00	3.03	0.08	67.29	15.33	0.18	0.04	0.43	0.20	0.02	9.78
S4	1.21	1.15	2.02	0.10	55.15	14.40	0.80	0.02	1.06	0.57	0.03	5.57
S5	0.52	0.28	1.38	0.02	51.61	8.63	1.78	0.01	0.93	0.32	0.05	0.40
S6	0.58	0.82	0.66	0.10	32.58	4.76	1.17	0.00	0.4	0.57	0.00	0.23
S7	0.44	0.56	1.48	0.06	41.61	10.85	1.70	0.02	1.2	0.45	0.04	0.35
S8	1.08	1.20	2.06	0.04	55.48	16.92	0.62	0.02	1.21	0.33	0.04	5.30
S9	0.48	0.68	0.00	0.05	49.65	13.97	0.48	0.02	0.77	0.36	0.00	3.24
S10	0.59	0.59	0.00	0.03	48.16	11.12	0.50	0.00	1.29	0.30	0.00	1.20
S11	0.66	0.73	1.06	0.00	38.85	9.19	2.93	0.07	2.56	0.71	0.29	0.46
S12	0.98	1.09	2.20	0.18	60.15	19.41	0.61	0.08	0.72	0.38	0.05	3.57
S13	0.31	0.43	1.50	0.50	55.40	9.73	1.04	0.02	0.72	0.38	0.11	0.86
S14	0.38	0.38	1.14	0.03	43.17	8.15	1.48	0.03	0.57	0.42	0.14	5.47
S15	0.25	0.37	1.21	0.02	45.62	8.39	2.15	0.03	0.93	0.64	0.15	0.07
S16	0.35	0.33	1.21	0.11	47.06	7.09	1.02	0.01	0.56	0.29	0.06	0.54
S17	0.06	0.08	0.00	0.05	24.54	3.69	1.29	0.03	0.58	0.66	0.13	0.70
S18	0.71	0.56	1.18	0.05	60.15	7.75	2.14	0.02	0.62	0.50	0.08	0.21
S19	0.39	0.50	1.71	0.04	54.53	10.59	1.61	0.05	0.65	0.64	0.12	4.77
S20	0.40	0.77	0.65	0.03	46.62	8.85	4.70	0.04	1.02	2.48	0.12	0.26
S21	0.41	0.45	1.43	0.16	64.64	9.61	8.37	0.08	0.61	0.86	0.14	0.33
S22	0.17	0.23	0.73	0.05	33.54	6.43	1.61	0.02	0.72	0.43	0.14	0.63
S23	0.16	0.24	1.02	0.07	41.63	7.59	12.04	0.12	0.86	1.59	0.23	0.79
S24	0.34	0.43	1.54	0.04	45.65	9.71	3.67	0.12	1.17	2.03	0.22	0.41
S25	1.02	0.24	3.16	0.08	73.09	3.82	0.62	0.17	0.97	0.70	0.02	0.60

## 4 DISCUSSION

GC–MS and HPLC approaches are of high sensitivity and accurate quantitative analysis method which are particularly suitable for rapidly analyzing phytochemical. EOs and flavonoids from CRP are the complex mixture of compounds with usage in health and medical sciences. In this work, all results suggested that the extraction method and GC–MS and HPLC analysis conditions applied to evaluate the quality comprehensively in 25 CRP samples collected from different districts in China were reliable. The variation in chemical compositions of the volatile oils and five bioactive flavonoids might be attributed to different geographic and environmental conditions (Chen & Cui, 1998). The chemical ingredients of volatile oil are not only an important source of spices, but also have a wealth of pharmacological activities, which are mainly composed of terpenoid, alcohols, and aldehydes from Citrus Reticulatae Pericarpium. Previous studies have shown that the extracts from CRP can be responsible for the antibacterial action, anticancer activity, insecticidal effect, and anti‐inflammatory and antioxidant ability in modern medicine. In addition, many studies show that terpenes have antifeedant activities, repellent actions, oviposition deterrent, contact toxicity activity, and fumigant activities in plant EOs (Xie, Yuan, Li, & Tang, 2001; Xu & Zhao, 1995; Xu, Zhao, Zhou, Ding, & Yu, 1994; Zhou et al., 2009). Plentiful of literatures concerning its efficacy in recent years have been published on a wide variety of articles. Moreover, chemical components may lead to different bioactive effects to some extent, especially the main bioactive compounds such as d‐limonene, α‐pinene, myrcene, and so on. In fact, the amounts of bioactive compounds play more important role in curing some diseases in TCMs used in China. A large and growing body of literature has investigated that the d‐limonene plays a significant role in antibacterial action, aroma enhancement function, and antitussive, expectorant, anti‐asthmatic, anti‐cancer, and other effects (Asamoto et al., 2002; Bezerra, Costa, & Nogueira, 2013; Huang, Sun, Long, & Sun, 2015; Shen, 2002). Significant antimicrobial activity of α‐terpineol has been proved (Cosentino et al., 1999). Previous studies have reported α‐pinene has antitussive, expectorant, antibacterial, and antifungal effects (Xia & Yu, 2000). There is a large volume of published studies describing the role of linalool has analgesic, anxiolytic, sedative hypnosis, anti‐inflammatory, antitumor, antibacterial, and other pharmacological activities (Jiang, Zhu, & Yu, 2015). Dried tangerine peel will possess great research value and broad market prospects of traditional Chinese medicine on account of a variety of pharmacological effects.

A wide variety of dried tangerine peels widely distributed throughout the country, whose medicine last for a long history. Profound traditional medicine, dried tangerine or orange peel, plays a significant role in curing respiratory system disease in China. Hence, it is necessary to further develop other medicinal value of orange peels owing to the insufficiency of viable *Citrus reticulata* Pericarpium. The China pharmacopeia (2015 version) has recorded the main cultivars of *C. reticulata* Blanco are *C. reticulata* “Chachi,” *C. reticulata* “Unshiu,” *C. reticulata* “Tangerina,” and *C. reticulata* “Dahongpao” in China. Our previous study has preliminarily investigated that *C. reticulata* “Ponkan” and *C. reticulata* “Shiyueju” generally are not applied to medicinal value as dried tangerine or orange peel possess a high amount of polymethoxylated flavones (PMFs), even the contents of those higher than the traditional famous region drug of GCP (Luo et al., 2017), similarly this experiment also found that the EO extraction yield and main chemical ingredients such as limonene of *C. reticulata* “Ponkan” and *C. reticulata* “Shiyueju” have come to the same conclusion.

## 5 CONCLUSION

In this study, a simple and sensitive GC–MS method provided a comprehensive analysis in aroma compositions and HPLC method was developed with five bioactive flavonoids contents in 25 batches of peel samples of 10 cultivars. The results obtained from this study provide a reference for developing other cultivars as a new type of medicinal resource to exert officinal value and health care value in the future.
